# MicroRNA expression profiles in human adipose-derived stem cells during chondrogenic differentiation

**DOI:** 10.3892/ijmm.2014.2051

**Published:** 2014-12-29

**Authors:** ZHEN YANG, JIE HAO, ZHEN-MING HU

**Affiliations:** Department of Orthopedic Surgery, The First Affiliated Hospital of Chongqing Medical University, Chongqing 400016, P.R. China

**Keywords:** bone morphogenetic protein receptor type 2, chondrogenesis, human adipose-derived stem cells, microRNA, miR-490-5p

## Abstract

The aim of the present study was to examine the microRNA (miRNA or miR) expression profiles during the chondrogenic differentiation of human adipose-derived stem cells (hADSCs) and identify the potential mechanisms through which miRNAs may affect the process of chondrogenesis. hADSCs were isolated and cultured. The expression levels of chondrogenic markers was detected by FACS analysis and immunohistochemistry. The miRNA expression profiles were then obtained through a miRNA array and confirmed through northern blot analysis. Putative targets of the miRNAs were predicted and validated through a luciferase reporter assay. The comparison of hADSCs following the induction of chondrogenic differentiation with undifferentiated hADSCs revealed 20 miRNAs that were differentially expressed by at least 2-fold, and these miRNAs included 12 upregulated miRNAs and 8 downregulated miRNAs. Northern blot analysis further confirmed the miRNA expression levels. Of these miRNAs, the expression of miR-490-5p was gradually downregulated following the induction of chondrogenic differentiation. The overexpression of miR-490-5p increased the expression of the chondrogenic markers, collagen, type II, alpha 1 (Col2A1), collagen, type X, alpha 1 (Col10A1) and aggrecan. Furthermore, it was confirmed that miR-490-5p directly targets bone morphogenetic protein receptor type 2 (BMPR2). In conclusion, in this study, we identified a set of miRNAs that may play key roles in the regulation of the chondrogenic differentiation of hADSCs. Our results may provide a basis for the further investigations into the molecular mechanisms of action of miRNAs in hADSC chondrogenesis.

## Introduction

MicroRNAs (miRNAs or miRs) are short non-coding RNAs (~22 nt long) that can repress translation by binding imperfectly to their target mRNA. The miRNAs are transcribed and processed by Drosha and Dicer and are then loaded into an RNA-induced silencing complex (RISC) that leads to the regulation of translation (1).

Available evidence suggests that miRNAs are involved in the regulation of a wide range of biological processes, including cell proliferation, apoptosis, cell differentiation and embryonic development (2–4). The important regulatory roles of miRNAs during chondrogenesis were recently identified. miR-92a has been reported to be highly enriched in chondrogenic progenitors, and it has been found that its inactivation stabilizes the mRNA expression of the bone morphogenetic protein (BMP) antagonist gene, noggin3, leading to the repression of BMP signaling and the abnormal function of chondrogenic progenitors, which results in the unsustainable survival of chondrogenic progenitors (5). The downregulation of miR-181b was identified during the chondrogenic differentiation of transforming growth factor (TGF)-β3-stimulated limb mesenchymal cells to negatively regulate chondrocyte differentiation by reducing matrix metalloproteinase (MMP)-13 expression and inducing the expression of type Ⅱ collagen (6). In a comparison of the changes occurring in miRNA expression levels during the chondrogenesis of mesenchymal stem cells (MSCs) *in vitro*, it was discovered that the expression of miR-140 was significantly altered (7). Furthermore, it was also discovered that miR-140 stimulated chondrogenesis *in vitro* by targeting Ras-related small GTPases (RALA) and thereby affecting SRY (sex determining region Y)-box (SOX)9 at the protein level (7). Another study on miR-140 demonstrated that equine cord blood-derived mesenchymal stromal cells expressed significantly higher levels of miR-140 after 14 days of chondrogenic differentiation (8). Furthermore, chemokine ligand 12 and disintegrin and metalloproteinase with thrombosponin motifs were verified as direct targets of miR-140 (8). The functional role of miR-23b was also found to be the induction of chondrogenic differentiation through the negative inhibition of protein kinase A (PKA) signaling (9).

However, the majority of previous studies have focused on miRNA expression profiles in bone marrow-derived MSCs or stromal cells (10–12). Human adipose-derived stem cells (hADSCs) display a differentiation capacity that shows their potential for use in regenerative medical and tissue engineering applications due to their easy accessibility, isolation and expandability to clinical scales in a comparatively short period of time (13,14). It has been demonstrated that the differentiation potential of hADSCs resembles that of MSCs. The similarities between these adult stem cells extend to the biochemical levels, including multiple surface proteins, such as CD29 and CD44 (10,15).

In this study, we examined the expression profiles of miRNAs during the chondrogenic differentiation of hADSCs using a miRNA microarray. The differentially expressed miRNAs between the undifferentiated hADSCs and chondrogenically differentiated hADSCs were verified by northern blot analysis. We then predicted their putative target genes through bioinformatics analysis and confirmed 1 miRNA target. The results of the present study provide new insight into the function of miRNAs during the chondrogenic differentiation of hADSCs.

## Materials and methods

### Isolation of hADSCs and induction of chondrogenic differentiation

For the isolation hADSCs, 3 samples of adipose tissue were obtained from donors who underwent elective liposuction or other abdominal surgery at the First Affiliated Hospital of Chongqing Medical University (Chongqing, China). All the donors provided written informed consent and signed approval forms, and the study was approved by the relevant ethics committee. Healthy hADSCs (passage 3) were harvested, resuspended in incomplete chondrogenic medium (basal medium) at a density of 2.5×10^7^ cells/ml, placed in a 24-well plate, and allowed to adhere at 37°C for 90 min. Subsequently, 500 ml of complete chondrogenic medium were added to each well. The complete chondrogenic medium (Cyagen Biosciences, Inc., Santa Clara, CA, USA) contained Dulbecco’s modified Eagle medium/mutrient mixture F-12 (DMEM/F-12), 5 ng/ml fibroblast growth factor (FGF)-2, 10 ng/ml TGF-β_1_, 50 *μ*g/ml Vc and 10^−7^ M dexamethasone. After 24 h of incubation, the cell droplets coalesced and became spherical. The complete medium was changed every 3 days.

The cell surface markers, CD29, CD44, CD49 and CD45, were detected using a flow cytometer (Beckman Coulter, Inc., Brea, CA, USA). The corresponding fluorescein isothiocyanate (FITC)-conjugated monoclonal mouse anti-human antibodies were as follows: CD29 (1:100, Cat. no. 119-15141; Raybiotech, Inc., Norcross, GA, USA), CD44 (1:100, Cat. no. 119-15548; Raybiotech, Inc.), CD49 (1:100, Cat. no. ABIN118708, antibodies-online.com, Aachen, Germany) and CD45 (1:100, Cat. no. 119-15144; Raybiotech, Inc.). The cells were incubated with the relevant antibodies at 4°C for 20 min, washed with phosphate-buffered saline (PBS), 0.1% NaN_3_ and 5% FBS, and then analyzed using a fluorescence-activated cell sorting (FACS) Calibur (Becton Dickinson, Franklin Lakes, NJ, USA). The data were analyzed using CellQuest3.1f software (Becton Dickinson).

### Immunohistochemical examination

hADSCs (passage 3) before and after the induction of chondrogenesis were subjected to immunohistochemical examination. The cells were washed 3 times with PBS and fixed with 95% alcohol for 10 min at room temperature. The cells were permeabilized with permeable solution for 10 min. After washing, endogenous peroxidase was quenched in the cells with 3% H_2_O_2_ in ethanol for 10 min, washed 3 times with Tris-buffered saline (TBS, pH 7.6), incubated with blocking solution for 10 min and then incubated with monoclonal mouse anti-human antibodies against collagen type II (ColII; ab3092; Abcam, Cambridge, UK) for 2 h. Subsequently, the cells were washed 3 times with TBS and incubated with a biotin-conjugated second antibody for 10 min. After washing with TBS, the cells were added to an enzyme (HRP streptavidin)-conjugated anti-biotin solution for 10 min and washed. The proteins were displayed using AEC mounting solution (Fuzhou Maixin Biotechnology Development Co., Ltd., Fuzhou, China) and then visualized and photographed.

### Total RNA extraction

Total RNA from the hADSCs prior to (controls) and after chondrogenic induction was isolated using the mirVana kit^®^ (Applied Biosystems, Foster City, CA, USA) according to the manufacturer’s instructions, and the concentration was determined by the ratio of the absorbance at 260 to that at 280 nm using a Nanodrop^®^ ND-1000 spectrophotometer (Thermo Scientific, Waltham, MA, USA).

### Western blot analysis

Western blot analysis was performed to determine the protein expression of BMPR2, SOX2 and GAPDH. Total protein from the cells were lysed by RIPA buffer. GAPDH was used as an endogenous normalizer. Polyclonal rabbit anti-human BMPR2 (1:1000, sc-20737; Santa Cruz Biotechnology, Santa Cruz, CA, USA), SOX2 (1:1000; sc-17319; Santa Cruz Biotechnology) and GAPDH (1:1000; sc-367714, Santa Cruz Biotechnology) antibodies were used.

### miRNA microarray analysis

After the RNA was isolated from the hADSCs prior to and after chondrogenic induction were compared, miRNA labeling was then performed using the miRCURY™ Hy3™/Hy5™ Power labeling kit (Exiqon, Vedbaek, Denmark). The Hy3^TM^ -labeled samples were then hybridized at 56°C overnight on a miRCURY™ LNA Array (v.16.0) (Exiqon), which contains probes for 1,223 human miRNAs, in a 12-Bay Hybridization System (Nimblegen Systems, Inc., Madison, WI, USA). Following hybridization, the slides were washed 5 times using a wash buffer kit (Exiqon) and dried. The slides were then scanned using a Axon GenePix 4000B microarray scanner (Axon Instruments, Foster City, CA, USA). We performed a fold change filtering to determine the differential expression of the miRNAs before and after the induction of the differentiation of the hADSCs in all 3 sets of hADSCs. The threshold used to screen the up- or down-regulated miRNAs was a fold change ≥2.0. Average linkage hierarchical clustering was performed to obtain clusters of data sets and generate a heatmap, using Gene Cluster and Treeview software (http://www.eisenlab.org/eisen/).

### Northern blot analysis of miRNA expression

Total RNA was isolated using TRIzol reagent (Invitrogen Life Technologies, Carlsbad, CA, USA) according to the manufacturer’s instructions. For the miRNA northern blot analyses, 15 *μ*g of the total RNA was separated on 15% denaturing polyacrylamide gels, electrotransferred to GeneScreen Plus membranes and hybridized using Ultra-Hyb-Oligo buffer (Ambion, Carlsbad, CA, USA) overnight at 42°C. Oligonucleotides complementary to the mature miRNAs were end-labeled with T4 kinase (Roche Applied Science, Indianapolis, IN, USA) and used as probes. The probe sequences were as follows: miR-196a antisense, 5′-CCCAACAACATGAAACTACCTA-3′; miR-193b antisense, 5′-AGCGGGACTTTGAGGGCCAGTT-3′; miR-383 antisense, 5′-AGCCACAATCACCTTCTGATCT-3′; miR-490-5p antisense, 5′-ACCCACCTGGAGATCCATGG-3′; miR-1307 antisense, 5′-AGCCGGTCGAGGTCCGGTCGA-3′; and U6 antisense, 5′-GCCATGCTAATCTTCTCTGTATC-3′.

### Bioinformatics analysis

The miRNA targets were predicted using the algorithms, TargetScan (www.targetscan.org), MiRanda (www.microrna.org) and miRBase Targets (microrna.sanger.ac.uk). The predicted targets were intersected using MatchMiner to identify the genes that were commonly predicted by the 3 different algorithms.

### Fluorescent reporter assays

The human BMP receptor type 2 (BMPR2) 3′ untranslated region (3′UTR) harboring the miR-490-5p target sequence and the seed sequence mutated version (BMPR2-3′UTR-mut) were synthesized by GenePharma (Shanghai, China) and then ligated after the luc ORF into the pMIR-REPORT luciferase vector (Ambion). For the fluorescent reporter assay, the cells were seeded in a 48-well plate and co-transfected with miR-490-5p mimics and BMPR2 3′UTR or BMPR2-3′UTR-mut. The cells were lysed 48 h after transfection, and the luciferase intensity was measured.

### Knockdown of PREX2a by siRNA

To knockdown the expression of BMPR2, a BMPR2 siRNA lentivirus was purchased from Applied Biological Materials, Inc. (Cat. no. i002001d; Richmond, BC, Canada). A scrambled siRNA GFP Lentivector was used as a control (Cat. no. LV015-G, Applied Biological Materials, Inc.).

### Enzyme-linked immunosorbent assay (ELISA)

The culture medium was collected at 12, 15, and 18 days following the induction of chondrogenic differentiation. ELISA was performed to detect the secreted concentrations of the chondrogenic markers, collagen, type II, alpha 1 (Col2A1), collagen, type X, alpha 1 (Col10A1) and aggrecan using specific ELISA assay kits (the ELISA kits were obtained from www.antibodies-online.com, and the catalog numbers were ABIN415072, ABIN1114237, ABIN1113295, respectively) according to the manufacturer’s instructions.

### Statistical analysis

The paired t-test was applied to examine the differences between the basal cell cultures and the cultures of hADSCs subjected to chondrogenic differentiation. A value of p<0.05 was considered to indicate a statistically significant difference. All the statistical analyses were two-sided and were carried out using the SPSS statistical software version 13.0 (SPSS, Inc., Chicago, IL, USA).

## Results

### Confirmation of chondrogenic differentiation of hADSCs

**Figure 1:**
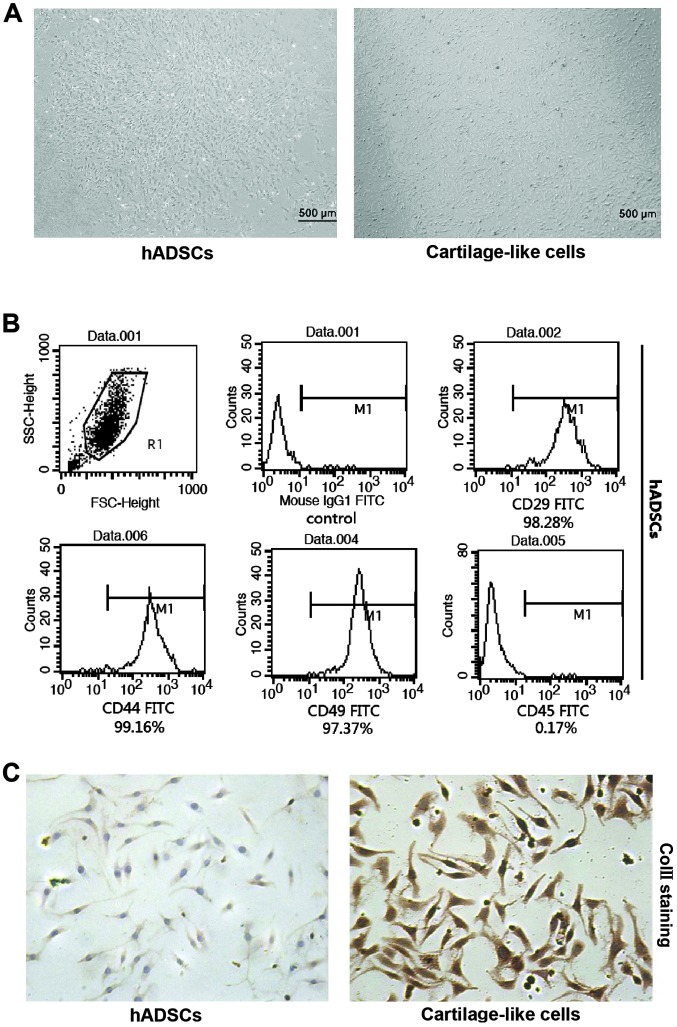
Confirmation of human adipose-derived stem cells (hADSCs) and the morphology of hADSCs subjected to chongrogenic differentiation. (A) Control hADSCs cultured in basal medium at passage 3 (left panel) and differentiated hADSCs on day 18 (right panel). (B) Approximately 98.28% of cells were positive for CD29, 99.16% were positive for CD44, 97.37% were positive for CD49 and only 0.17% were positive for CD45 (almost negative in the isolated hADSCs). (C) The expression level of collagen type II (ColII) was significantly higher in the hADSCs subjected to chongrogenic differentiation compared with the cells cultured in basal medium.

hADSCs at passage 3, which included small, round individual or several cells clumped together suspended in medium, were cultured in basal or chondrogenic induction medium for 18 days. In the basal medium, the cells were tightly packed (Fig. 1A, left panel), whereas the morphology of the cells cultured in the chondrogenic induction medium was altered and consisted of polygon-shaped cells, which is a typical characteristic of chondrocyte morphology, as shown in Fig. 1A (right panel). The cell surface markers in the cultured cells were detected by flow cytometry at passage 3 following incubation with FITC-labeled antibodies. In accordance with the antigenic profiles of hADSCs reported previously (15,16,20), the cells were positive for CD29, CD44 and CD49 and negative for CD45 (Fig. 1B). To ascertain whether the hADSCs had undergone chondrogenic differentiation in the chondrogenic induction medium, the expression of proteins associated with chondrogenesis was detected by immunohistochemical examination (Fig. 1C). Following culture in chondrogenic induction medium for 18 days, the level of ColII in the cells subjected to chondrogenic differentiation was significantly higher than that in the cells cultured in basal medium. These results provide evidence of chondrogenic differentiation in the cells exposed to the chondrogenic induction medium.

### Analysis of miRNA expression before and after the induction of chondrogenic differentiation

**Figure 2:**
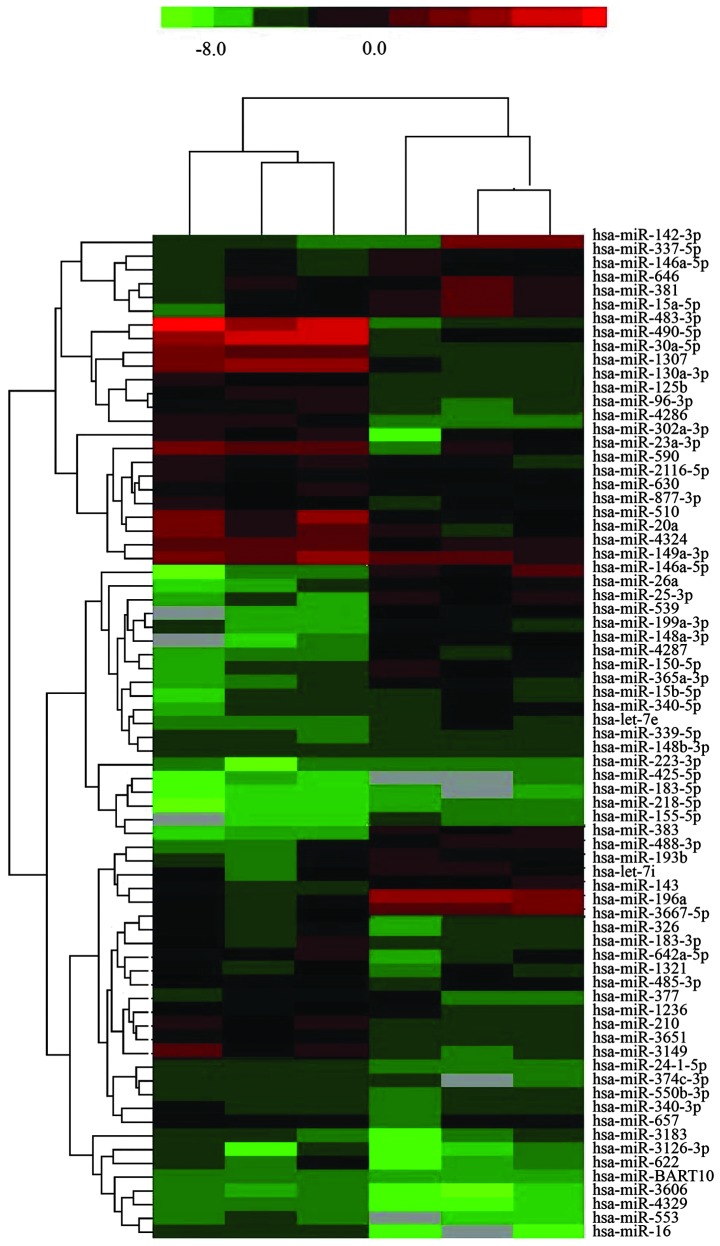
Expression profiles of miRNAs in human adipose-derived stem cells (hADSCs) cultured with or without chondrogenic induction medium. Heatmap illustrates the levels of miRNAs with a significant change in expression [fold change >2 and false discovery rate (FDR) <0.0001%]. Color intensity is scaled within each row, such that the highest expression value corresponds to bright red and the lowest to bright green.

The miRNA expression levels of hADSCs before and after the induction of chondrogenic differentiation were detected using miRNA microarray chips. The miRNAs that exhibited a differential expression between the undifferentiated hADSCs and the hADSCs subjected to chondrogenic differentiation were identified (Fig. 2). The miRNAs that exhibited at least a 2-fold change in expression in the hADSCs before and after the induction of chondrogenic differentiation are listed in Table I, and these include 12 upregulated miRNAs (miR-196a, miR-143, miR-383, miR-193b, let-7i, miR-26a, miR-539, miR-199a-3p, miR-337-5p, miR-146a-5p, miR-646, and miR-381) and 8 downregulated miRNAs (miR-490-5p, miR-1307, miR-125b, miR-96-3p, miR-302-3p, miR-23a-3p, miR-590, and miR-510).

### Validation of the microarray results by northern blot analysis

**Figure 3:**
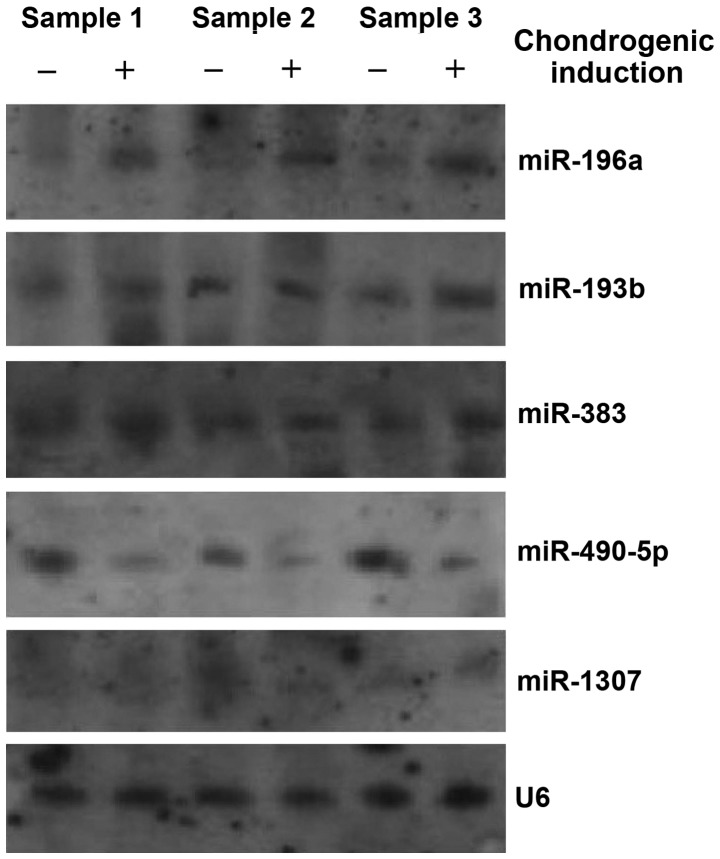
Northern blot analysis confirmation of miRNA expression in human adipose-derived stem cells (hADSCs) isolated from 3 sets of samples subjected to chondrogenic differentiation. Total RNA (15 *μ*g) was separated on 15% denaturing polyacrylamide gels, electrotransferred to GeneScreen Plus membranes, and hybridized with miRNA oligo-nucleotides end-labeled with T4 kinase.

In order to confirm the results of the microarray analysis, we conducted a northern blot analysis to detect the expression levels of 8 representative differentially expressed miRNAs identified using the microarray, including 4 upregulated miRNAs (miR-196a, miR-193b, miR-383 and miR-143) and 4 downregulated miRNAs (miR-490-5p, miR-1307, miR-125b and miR-590). However, only 5 miRNAs [miR-196a, miR-193b, miR-383 (upregulated), miR-490-5p and miR-1307 (downregulated)] produced northern blot analysis signals. The results from northern blot analysis also revealed that miR-196a was overexpressed in all the 3 samples, whereas miR-193b and miR-383 were overexpressed in only samples 1 and 3 between the undifferentiated hADSCs and the hADSCs which were subjected to chondrogenic differentiation. Of the 2 downregulated miRNAs, miR-490-5p was significantly downregulated in all 3 samples, and the expression of miR-1307 was down-regulated only in sample 2 (Fig. 3).

### Effect of miR-490-5p on chondrogenic differentiation of hADSCs

**Figure 4:**
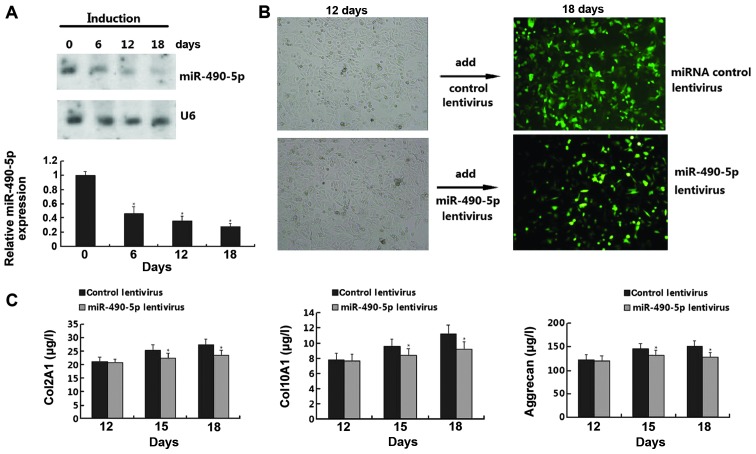
Role of miR-490-5p in the chondrogenic differentiation of human adipose-derived stem cells (hADSCs). (A) The expression of miR-490-5p was detected by northern blot analysis following the induction of chondrogenic differentiation on days 0, 6, 12 and 18. (B) Cell morphology was determined following transfection with miR-490-5p letivirus on days 12 and 18. (C) Comparison of the concentration of collagen, type II, alpha 1 (Col2A1), collagen, type X, alpha 1 (Col10A1) and aggrecan in hADSCs subjected to chondrogenic differentiation on days 12, 15 and 18 by ELISA.

Since we confirmed that miR-196a and miR-490-5p were overexpressed and downregualted, respectively, in all 3 samples, we conducted functional analysis to detect the role of the 2 miRNAs in the chondrogenic differentiation of hADSCs. However, we only found that miR-490-5p, but not miR-196a (data not shown), had an obvious effect on chondrogenic differentiation. As shown in Fig. 4A, the chondrogenic differentiation of the hADSCs resulted in the downregulation of miR-490-5p in a time-dependent manner. However, when the cells were transfected with miR-490-5p lentivirus on day 12, the cell morphology was reversed on day 18 compared with the control lentivirus group. In order to further confirm the results that miR-490-5p regulates the chondrogenic differentiation of hADSCs, we performed an ELISA for the quantitative measurement of chondrogenic differentiation markers (Col2A1, Col10A1 and aggrecan); the levels of these markers were gradually upregulated on days 12, 15, and 18; however, the levels of these markers were downregulated in the cells transfected with the miR-490-5p lentivirus (Fig. 4C). Thus, these results demonstrate that miR-490-5p inhibits the chondrogenic differentiation of hADSCs.

### BMPR2 as a direct target gene of miR-490-5p

**Figure 5:**
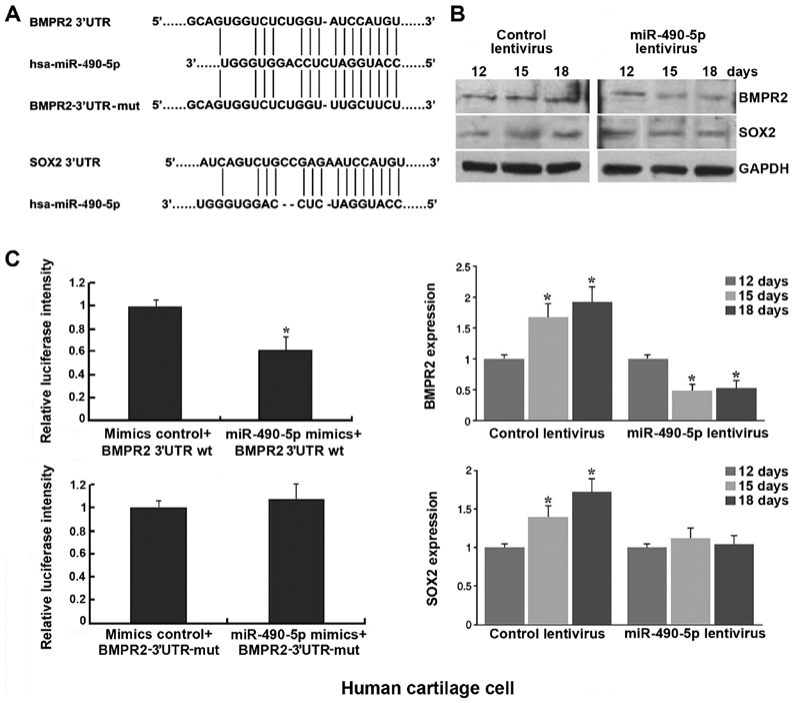
miR-490-5p directly targets bone morphogenetic protein receptor type 2 (BMPR2) and inhibits its expression. (A) A schematic of the bioinformatics predicted seed region in the 3′UTR of BMPR2 as well as mutated 3′UTR used in this study. (B) Effect of transfection with miR-490-5p lentivirus on the protein level of BMPR2 in human adipose-derived stem cells (hADSCs) subjected to chondrogenic differentiation on days 12, 15 and 18. (C) Comparison of luciferase activity of cells transfected with BMPR2-3′UTR and BMPR2-3′UTR-mut and cells transfected with miR-490-5p mimics and miR-490-5p control. ^*^P<0.05.

Bioinformatics analysis identified SOX2 and BMPR2 as putative targets of miR-490-5p (Fig. 5A), which are related to chondrogenic differentiation. A western blot analysis was then performed to examine the protein expression of the predicted targets SOX-2 and BMPR2. The results revealed that the expression levels of SOX2 and BMPR2 in the hADSCs subjected to chondrogenic differentiation were upregulated, and the transfection of the cells with miR-490-5p lentivirus resulted in the downregulation of BMPR2 after the induction of hADSC differentiation, whereas the expression of SOX2 was not obviously affected (Fig. 5B). Furthermore, luciferase reporter assays confirmed that miR-490-5p suppressed the BMPR2 3′UTR luciferase activity by 40%, and the luciferase activity was completely reversed following transfection with mutated BMPR2 3′UTR (Fig. 5C). Therefore, we confirmed that BMPR2 is a direct target of miR-490-5p.

### Knockdown of BMPR2 inhibits the hADSC chondrogenic differentiation

**Figure 6:**
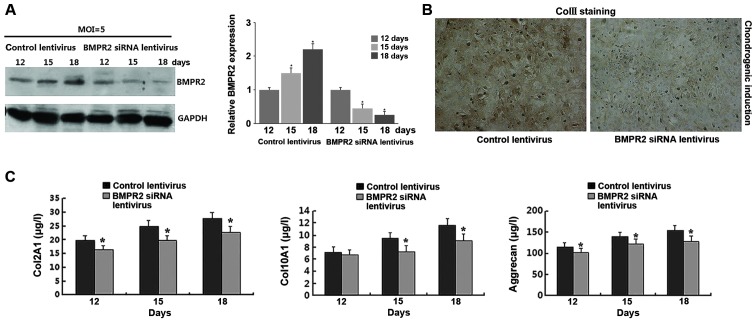
Functional role of bone morphogenetic protein receptor type 2 (BMPR2) in the chondrogenic differentation of human adipose-derived stem cells (hADSCs). (A) hADSCs were infected with BMPR2 siRNA lentivirus and the expression of BMPR2 protein expression was detected following the induction of chondrogenic differentation on days 12, 15 and 18. (B) The expression level of collagen type II (ColII) was detected with immunohistochemical analysis on day 18 after the induction of chondrogenic differentation. (C) Cells were infected with BMPR2 siRNA lentivirus and the concentration of collagen, type II, alpha 1 (Col2A1), collagen, type X, alpha 1 (Col10A1) and aggrecan was detected following the induction of chondrogenic differentation on days 12, 15 and 18 by ELISA.

To investigate the functional role of BMPR2 in the chondrogenic differentiation of hADSCs, BMPR2 siRNA lentivirus was transfected into the hADSCs and this resulted in a significant decrease in BMPR2 protein expression in a time-dependent manner following the induction of chondrogenic differentiation (Fig. 6A). The immunohistochemical examination illustrated that ColII was downregulated in the cells transfected with BMPR2 siRNA lentivirus compared with the cells transfected with the control lentivirus after the induction of chondrogenic differentiation for 18 days (Fig. 6B). Consistent with the effect of miR-490-5p, we used ELISA analysis and found that the levels of Col2A1, Col10A1 and aggrecan were downregulated on days 12, 15 and 18 in the cells transfected with BMPR2 siRNA lentivirus (Fig. 6C).

## Discussion

The use of stem cells for cartilage regeneration is hampered by a lack of knowledge of the molecular mechanisms of chondrogenesis, which require the stringent control of a program for gene activation and suppression in response to biological cues (17,18). Due to the complexity of the biological network associated with cellular differentiation, the identification of regulatory miRNAs and their direct targets is valuable (19). Although the functions of some individual miRNAs in chondrogenesis are known, the mechanisms through which a set of miRNAs regulate the onset of a tissue-specific phenotype remain ambiguous (20). The analysis of miRNA expression profiles may substantially help to determine the mechanisms responsible for chondrocyte development and may eventually lead to the development of therapeutic interventions (21).

In support of the hypothesis that miRNAs play a key role in chondrogenesis, in this study, we investigated chondro-miRNAs to provide insight into the specific involvement of miRNAs in the chondrogenesis of hADSCs. We used the miRNA microarray technique to determine the miRNA expression profiles and screen miRNAs with a significant change in expression (>2-fold) before and after the induction of chondrogenic differentiation. A northern blot analysis was conducted to confirm that miR-196a and miR-490-5p were indeed differentially expressed in all 3 samples, which is consistent with the microarray results, whereas miR-193b and miR-383 were found to be upregulated in only 2 samples, and miR-1307 was downregulated in only 1 sample. Our findings on the reduction in the expression of miR-490-5p were in line with those of a previous study (22).

Since miR-196a and miR-490-5p were significantly differentially expressed in all 3 samples, we conducted a functional analysis. However, we found that miR-490-5p, but not miR-196a (data not shown), had an obvious effect on chondrogenic differentiation. Accompanying hADSC differentiation, the expression of miR-490-5p was gradually downregulated and transfection with miR-490-5p lentivirus reversed the differentiation ability of the hADSCs. Using a luciferase reporter system, miR-490-5p was proven to target the 3′UTR of BMPR2. Thus, miR-490-5p inhibits hADSC differentiation by suppressing BMPR2 expression.

The confirmation of proper chondrogenesis is usually determined by an analysis of ‘end-stage’ differentiation markers, such as Col2A1, Col10A1 and aggrecan (23–25). The strongest upregulation of the proteins was observed in the hADSCs subjected to chondrogenic differentiation, which confirmed that the hADSCs had differentiated into chondrocytes. Moreover, we identified that miR-490-5p lentivirus partly rescued the expression of these markers, and the downregulation of BMPR2 by siRNA lentivirus had similar effects as the overexpression of miR-490-5p, which further indicates that miR-490-5p inhibits the process of chondrogenesis by targeting BMPR2.

In conclusion, we discovered a set of miRNAs that are differentially expressed during the process of hADSC chondrogenic differentiation and confirmed that miR-490-5p has the potential to inhibit the chondrogenesis of hADSCs. To enhance our understanding of the regulatory mechanisms of these differentially expressed miRNAs during chondrogenic differentiation, further studies are required. Our results shed new light for further investigation into the molecular mechanisms of the chondrogenic differentiation of hADSCs.

## Figures and Tables

**Table I:** Microarray analysis of average expression of miRNAs in hADSCs isolated from 3 sets of samples subjected to chondrogenic differentiation.

miRNA	Gene ID	Average fold change	p-value
miR-196a	406972	6.87	0.0021
miR-143	406935	4.52	0.0038
miR-383	494332	5.16	0.0149
miR-193b	574455	5.62	0.0057
let-7i	406891	3.41	0.0025
miR-26a	407015	3.72	0.0031
miR-539	664612	2.39	0.0041
miR-199a-3p	406976	3.19	0.0131
miR-337-5p	442905	2.81	0.0093
miR-146a-5p	406938	2.16	0.0071
miR-646	693231	3.05	0.0049
miR-381	494330	2.47	0.0051
miR-490-5p	574443	0.13	0.0017
miR-1307	100302174	0.38	0.0081
miR-125b	406911	0.29	0.0038
miR-96-3p	407053	0.41	0.0217
miR-302-3p	407028	0.47	0.0029
miR-23a-3p	407018	0.35	0.0024
miR-590	693175	0.23	0.0036
miR-510	574515	0.35	0.0077

hADSCs, human adipose-derived stem cells.

## References

[1] Dong S, Yang B, Guo H, Kang F (2012). MicroRNAs regulate osteogenesis and chondrogenesis. Biochem Biophys Res Commun.

[2] Huang J, Zhao L, Xing L, Chen D (2010). MicroRNA-204 regulates Runx2 protein expression and mesenchymal progenitor cell differentiation. Stem Cells.

[3] Tomé M, López-Romero P, Albo C, Sepúlveda JC, Fernández-Gutiérrez B, Dopazo A, Bernad A, González MA (2011). miR-335 orchestrates cell proliferation, migration and differentiation in human mesenchymal stem cells. Cell Death Differ.

[4] Wagner W, Horn P, Castoldi M, Diehlmann A, Bork S, Saffrich R, Benes V, Blake J, Pfister S, Eckstein V, Ho AD (2008). Replicative senescence of mesenchymal stem cells: a continuous and organized process. PloS One.

[5] Ning G, Liu X, Dai M, Meng A, Wang Q (2013). MicroRNA-92a upholds Bmp signaling by targeting noggin3 during pharyngeal cartilage formation. Dev Cell.

[6] Song J, Lee M, Kim D, Han J, Chun CH, Jin EJ (2013). MicroRNA-181b regulates articular chondrocytes differentiation and cartilage integrity. Biochem Biophys Res Commun.

[7] Karlsen TA, Jakobsen RB, Mikkelsen TS, Brinchmann JE (2014). microRNA-140 targets RALA and regulates chondrogenic differentiation of human mesenchymal stem cells by translational enhancement of SOX9 and ACAN. Stem Cells Dev.

[8] Buechli ME, Lamarre J, Koch TG (2013). MicroRNA-140 expression during chondrogenic differentiation of equine cord blood-derived mesenchymal stromal cells. Stem Cells Dev.

[9] Ham O, Song BW, Lee SY, Choi E, Cha MJ, Lee CY, Park JH, Kim IK, Chang W, Lim S, Lee CH, Kim S, Jang Y, Hwang KC (2012). The role of microRNA-23b in the differentiation of MSC into chondrocyte by targeting protein kinase A signaling. Biomaterials.

[10] Bossio C, Mastrangelo R, Morini R, Tonna N, Coco S, Verderio C, Matteoli M, Bianco F (2013). A simple method to generate adipose stem cell-derived neurons for screening purposes. J Mol Neurosci.

[11] Pandey AC, Semon JA, Kaushal D, O’Sullivan RP, Glowacki J, Gimble JM, Bunnell BA MicroRNA profiling reveals age-dependent differential expression of nuclear factor κB and mitogen-activated protein kinase in adipose and bone marrow-derived human mesenchymal stem cells. Stem Cell Res Ther.

[12] Crobu F, Latini V, Marongiu MF, Sogos V, Scintu F, Porcu S, Casu C, Badiali M, Sanna A, Manchinu MF, Ristaldi MS (2012). Differentiation of single cell derived human mesenchymal stem cells into cells with a neuronal phenotype: RNA and microRNA expression profile. Mol Biol Rep.

[13] Guan L, Shaoqing L, Wang Y, Yue H, Liu D, He L, Bai C, Yan F, Nan X, Shi S, Pei X (2006). In vitro differentiation of human adipose-derived mesenchymal stem cells into endothelial-like cells. Chinese Science Bulletin.

[14] Zhu Y, Liu T, Song K, Fan X, Ma X, Cui Z (2008). Adipose-derived stem cell: a better stem cell than BMSC. Cell Biochem Funct.

[15] Peroni D, Scambi I, Pasini A, Lisi V, Bifari F, Krampera M, Rigotti G, Sbarbati A, Galiè M (2008). Stem molecular signature of adipose-derived stromal cells. Exp Cell Res.

[16] Gronthos S, Franklin DM, Leddy HA, Robey PG, Storms RW, Gimble JM (2001). Surface protein characterization of human adipose tissue-derived stromal cells. J Cell Physiol.

[17] Chen FH, Rousche KT, Tuan RS (2006). Technology Insight: adult stem cells in cartilage regeneration and tissue engineering. Nat Clin Pract Rheumatol.

[18] Vilquin JT, Rosset P (2006). Mesenchymal stem cells in bone and cartilage repair: current status. Regen Med.

[19] Iliopoulos D, Malizos KN, Oikonomou P, Tsezou A (2008). Integrative microRNA and proteomic approaches identify novel osteoarthritis genes and their collaborative metabolic and inflammatory networks. PLoS One.

[20] Han J, Yang T, Gao J, Wu J, Qiu X, Fan Q, Ma B (2010). Specific microRNA expression during chondrogenesis of human mesenchymal stem cells. Int J Mol Med.

[21] Bakhshandeh B, Soleimani M, Paylakhi SH, Ghaemi N (2012). A microRNA signature associated with chondrogenic lineage commitment. J Genet.

[22] Zhang Z, Kang Y, Zhang Z, Zhang H, Duan X, Liu J, Li X, Liao W (2012). Expression of microRNAs during chondrogenesis of human adipose-derived stem cells. Osteoarthritis Cartilage.

[23] Indrawattana N, Chen G, Tadokoro M, Shann LH, Ohgushi H, Tateishi T, Tanaka J, Bunyaratvej A (2004). Growth factor combination for chondrogenic induction from human mesenchymal stem cell. Biochem Biophys Res Commun.

[24] Ronzière MC, Perrier E, Mallein-Gerin F, Freyria AM (2010). Chondrogenic potential of bone marrow- and adipose tissue-derived adult human mesenchymal stem cells. Biomed Mater Eng.

[25] Zhao Q, Eberspaecher H, Lefebvre V, De Crombrugghe B (1997). Parallel expression of Sox9 and Col2a1 in cells undergoing chondrogenesis. Dev Dyn.

